# Supplementary material for: Gamification as an Educational Approach for Oncological Patients: A Systematic Scoping Review
Source: Healthcare (Basel). 2023 Dec 7;11(24):3116. doi: 10.3390/healthcare11243116 (PMC10742971; doi:10.3390/healthcare11243116)
Supplement: Supplementary file 1 [file healthcare-11-03116-s001.zip › Table S1- Search strategies.pdf]

Table S1: Search strategies

MEDLINE with PubMed

("Neoplasms"[MeSH Terms] OR "Neoplasms"[Title/Abstract] OR "cancer"[Title/Abstract] OR "tumour"[Title/Abstract] OR "tumor"[Title/Abstract] OR "Medical Oncology"[Mesh] OR "Surgical Oncology"[Mesh] OR “oncology” OR “medical oncology” OR “surgical oncology” OR "Neoplasm Metastasis"[Mesh] OR “neoplasm metastasis” OR “metastasis” OR “metastatic” OR “metastases” OR “oncology patients” OR “cancer patients” OR “patients with cancer” OR "Cancer Survivors"[Mesh] OR “cancer survivor*”) AND ("Gamification"[MeSH] OR "Gamification"[Title/Abstract] OR "serious gam*"[Title/Abstract] OR "gamif*" [Title/Abstract] OR “game-based learning” [Title/Abstract])

EMBASE [Elsevier]

(cancer'/exp OR 'cancer':ti,ab OR 'tumour'/exp OR 'tumour':ti,ab OR 'tumor'/exp OR 'tumor':ti,ab OR 'oncology'/exp OR 'oncology':ti,ab OR 'neoplasm'/exp OR 'neoplasm':ti,ab OR 'medical oncology'/exp OR 'medical oncology':ti,ab OR 'surgical oncology'/exp OR 'surgical oncology':ti,ab OR 'neoplasm metastasis'/exp OR 'neoplasm metastasis':ti,ab OR 'metastasis'/exp OR 'metastasis':ti,ab OR 'metastatic':ti,ab OR 'metastases'/exp OR 'metastases':ti,ab OR 'oncology patients':ti,ab OR 'cancer patients':ti,ab OR 'patients with cancer':ti,ab OR 'cancer survivors'/exp OR 'cancer survivors':ti,ab) AND ('gamification'/exp OR 'gamification':ti,ab OR 'serious game'/exp OR 'serious gam*':ti,ab OR 'gamif*':ti,ab OR 'game-based learning':ti,ab)

PsycInfo [EBSCO]

((MM "Neoplasms") OR TI"neoplasms" OR AB"neoplasms" OR TI"cancer" OR AB"cancer" OR TI"tumour" OR AB"tumour" OR TI"tumor" OR AB"tumor" OR TI“medical oncology” OR AB“medical oncology” OR TI“surgical oncology” OR AB“surgical oncology” OR TI“oncology” OR AB“oncology” OR TI“neoplasm metastasis” AB“neoplasm metastasis” OR TI“metastasis” OR AB“metastasis” OR TI“metastatic” OR AB“metastatic” OR TI“metastases” OR AB“metastases” OR TI“oncology patients” OR AB“oncology patients” OR TI“cancer patients” OR AB“cancer patients” OR TI“patients with cancer” OR AB“patients with cancer“ OR TI“cancer survivor” OR AB“cancer survivor” OR (MM “Metastasis”) OR (MM “Oncology”)) AND (TI"gamification" OR AB"gamification" OR TI"serious gam*" OR AB"serious gam*" OR TI“gamif*” OR AB“gamif*” OR TI“game-based learning” OR AB“game-based learning”)

CINAHL [EBSCO]

Query	Limiters/Expanders	Results
**S10**	S8 AND S9	8
**S9**	S2 OR S3	530
**S8**	S1 OR S4 OR S5 OR S6 OR S7	707,487
**S7**	(MM "Cancer Survivors") OR (MM "Cancer Patients")	36,474
**S6**	(MH "Oncology+")	12,780
**S5**	(MH "Neoplasms+")	650,613
**S4**	(MH "Neoplasm Metastasis+")	45,322
**S3**	(MM "Gamification")	149
**S2**	TI (("gamification" OR "serious gam*" OR "gamif*" OR "game-based learning")) AND AB (("gamification" OR "serious gam*" OR "gamif*" OR "game-based learning"))	488
**S1**	TI (("neoplasms" OR "cancer" OR "tumour" OR "tumor" OR "oncology" OR "oncologic patient" OR "surgical oncology" OR "medical oncology" OR "cancer survivor" OR "metastasis" OR "metastatic" OR "metastases")) AND AB (("neoplasms" OR "cancer" OR "tumour" OR "tumor" OR "oncology" OR "oncologic patient" OR "surgical oncology" OR "medical oncology" OR "cancer survivor" OR "metastasis" OR "metastatic" OR "metastases"))	233,497

Scopus [Elsevier]

TITLE-ABS-KEY (("gamification" OR "serious gam*" OR "gamif*" OR "game-based learning") AND ("neoplasms" OR "cancer" OR "tumour" OR "tumor" OR "oncology" OR "oncologic patient" OR "surgical oncology" OR "medical oncology" OR "cancer survivor" OR "metastasis" OR "metastatic" OR "metastases"))

The Cochrane Library

Query	Limiters/Expanders	Results
**#10**	#6 AND #9	22
**#9**	#7 OR #8	535
**#8**	("gamification" OR "serious game" OR “game-based learning”)	535
**#7**	MeSH descriptor: [Gamification] explode all trees	14
**#6**	#1 OR #2 OR #3 OR #4 OR #5	234902
**#5**	(("neoplasms" OR "cancer" OR "tumour" OR "tumor" OR “medical oncology” OR “surgical oncology” OR “oncology” OR “neoplasm metastasis” OR “metastasis” OR “metastatic” OR “metastases” OR “oncology patients” OR “cancer patients” OR “patients with cancer” OR “cancer survivors”)):ti,ab,kw with Cochrane Library publication date Between Jan 2000 and Jan 2022 (Word variations have been searched)	206749
**#4**	MeSH descriptor: [Neoplasms] explode all trees	90265
**#3**	MeSH descriptor: [Cancer Survivors] explode all trees	540
**#2**	MeSH descriptor: [Surgical Oncology] explode all trees	3
**#1**	MeSH descriptor: [Medical Oncology] explode all trees	269
