# Supplementary material for: Gamification as an Educational Approach for Oncological Patients: A Systematic Scoping Review
Source: Healthcare (Basel). 2023 Dec 7;11(24):3116. doi: 10.3390/healthcare11243116 (PMC10742971; doi:10.3390/healthcare11243116)
Supplement: Supplementary file 1 [file healthcare-11-03116-s001.zip › Table S2- Data extraction Table.pdf]

Table S2: Data extraction table

Authors Year	Publication Type	Country	Topic	Aim	Population	Methods	Intervention	Outcomes	Game elements	Key findings	Limits
Carcioppolo N.; Kim S.; Sanchez M.; Mao B.; Malova E.; Ryan A.; Lun D; Ewing C.; Hu S. (2022)	Online randomized experiment	USA	Dermatology	To develop and evaluate an online, game-based melanoma identification training intervention	1205 participants completed all three steps of the study. All participants were adults. Median age=47.31, 53% were female, and most of the population has a bachelor's degree or graduate/professional degree (28.8%/20.0%)	Online randomised experiment tests, four training types (ABCD, UDS, ABCD-F, control) and three feedback types (dermatological, dermatological + motivational, and control). Primary outcome: melanoma identification accuracy. Secondary outcomes: skin cancer prevention, attitudes, and prevention intentions. Three waves of the study: pre-screening survey, melanoma identification game, and post-test survey.	twelve game conditions in which the avatar is a detective mole (the animal) that should distinguish the healthy moles (swipe left) from the moles with melanoma (swipe right). First round was a training round receiving material: ABCD, UDS, ABCD-F or no training (control)	Identification and prevention of melanoma	training, real time feedback, avatar (mole), artwork and storyline, structure of tinder (swipe left or right)	ABCD and UDS training increase skin cancer awarness, prevention in patient with high risk or developing melanoma and with previous melanoma diagnosis	Sample from U.S. population level. Majority of hispanic Latino population in the study's area
Horsham C.; Dutton-Regester K.; Antrobus J.; Goldston A.; Price H.; Ford H.; Hacker E. (2021)	Two-phase design approach: development and qualitative analysis	Australia	Dermatology	To develop a virtual reality game containing preventive skin cancer messaging (focus on sun protection) and to assess the safety and satisfaction of the design based on end user feedback.	18 australian voluntaries partecipated to virtual reality games and focus groups (each ranged from 1-4 participants). Participants' characteristics: university educated 72%, female 67%, had never experienced VR before 61%, aged 18 -74 years, with sun-sensitive characteristics (more than half of participants). Overall, 2 out of 18 (11%) participants had previously been diagnosed with skin cancer (both basal cell carcinoma).	Two-phase design approach: game design (phase 1) and prototype testing (phase 2). Participants were recruited via university emails and the social media platform Facebook during September (spring) in Australia. Use of web-based demographic survey and a follow-up survey 7 days after the VR experience. Focus group or interview based on VR. The sun protection habits index was used.	60-minute focus group or interview, testing the 3- to 5-minute virtual reality experience and providing feedback. The participant performs a first-person shooter in the game, in the setting of the body, for example, the lung. The shooter must fight against cancer growth through weapons (chemotherapy, targeted therapy, and immunotherapy), facing quick proliferation and cell resistance. ABC Framework: appeal, belong, commitment.	Satisfaction with the experience, facilitate information understanding, empowerment in taking responsibility, change of behaviour, motivation in improving sun protection habits	Levels, different scenarios, shooter game, voice-over, audio-describing, virtual reality experience, time pressure, competition	Females tended to report more frequent use of sunglasses and sunscreen on their faces, whereas men reported the use of long sleeves and limiting time outdoors during midday hours. Increased sun protection behaviours among participants > 36 years compared with younger. 11% refers nausea during VR experience. Empowered participants take responsibility for their sun protection behaviors and they were motivated in improving sun protection habits in the future	Convenience sample, mostly female, and highly educated, which may limit the generalizability of the study findings. Cost and worries about the risk of infections by sharing the VR equipment.

Kim S.M., Kim H., Hwang H.C., Hong J.S., Bae S., Min K.J., Han D.H. (2018)	Pilot study	Korea	Breast Cancer	To investigate the effects of a serious game on depressive symptoms in breast cancer patients with depression using clinical scales measures and analysing Resting-State Networks.	30 finished the study, clinical stage I-IV female breast cancer patients with depressive moods. All adults with a diagnosis of mild to moderate MDD were included. (The diagnosis was conducted based on a score range from 10 to 29 on the Beck Depression Inventory). Only right-handed people were included.	35 participants were randomly classified into the game group (n=18) and the Non-game group (n=17). Game group (N=18) and the Non-game group (N=17). During the study period, all participants briefly met psychiatrists once a week to screen for aggravation of depression. After enrollment screening, all participants were assessed with the BDI (Beck Depression Inventory) ,28 Beck Anxiety Inventory (BAI),29 a modified form of the Stress Response Inventory (SRI),30,31 and fMRI. The BDI and BAI are self-report measures of the severity of depression and anxiety, respectively. The SRI is another self-rating scale regarding participant stress responses. Participants have to play the game for 3 weeks. At the end of 3 weeks, we repeated BDI, BAI, and SRI assessments and fMRI.	For three weeks, participants in the Game group were asked to play the serious game ''Hit the Cancer'' for at least 30 minutes/day and 5 days/week for 3 weeks. The game platform automatically recorded participant game playing time. Participants in the Non-game group received typical care for 3 weeks.	Functional Connectivity (FC) between the right posterior cingulate cortex (PCC) of the Default Mode Network (DMN) and the right insula of the Salience Network (SN). FC was measured with a functional MRI. Depression was measured with Beck Depression Inventory and Stress Response Inventory (SRI), and anxiety with Beck Anxiety Inventory.	"HIT the CANCER" (RAW HAND, Seoul, Korea). Third-person shooter game in the serious games genre. Game players are asked to selectively remove the cancer cells, with a higher score achieved for removal of a large number of cancer cells	Results suggest that 3 weeks of serious gameplay improved depressive symptoms and stress levels in breast cancer patients with depression. Playing a serious game produced a change in FC between the right insula of the SN and the right PCC of the DMN. In addition, there was an association between improvement in depressive symptoms and stress levels and a change in FC between the right insula of the SN and right PCC of the DMN. Improve selective attention, reduced stress level The serious game seemed to be effective by disengaging self-referential thought and negative rumination by training for enhanced function in modulating the switch between self-focused thinking and goal-directed cognitive processes.	Short study period: 3 weeks. The study did not assess the usability of the game in cancer patients. A small number of participants. Heterogeneity of anticancer drugs. Some anticancer drugs could develop neurotoxic effects.
Loerzel V., Clochesy J., Geddie P. (2018)	Community Advisory Board (CAB)	USA	CINV	To develop a relevant, age-appropriate, tailored intervention for older adults to teach and reinforce how to self-manage CINV better at home.	older adults in treatment for cancer (n=5), caregivers (n=3), oncology nurses (n=4)	3 focus groups (semistructured interviews were conducted one-to-one). Interviews were conducted during chemotherapy sessions (patient and caregiver) and at the end of the work shifts (nurses). The first interview was centred on the experience of CINV, the second on validating themes and providing feedback on the game prototype and the last was based on final thoughts and play testing.	Gamed conversation among avatars in which the patient provides information about nausea and vomiting to the caregiver. Information: what they ate and drank when nauseated, how they treated themselves when feeling good, what they did to manage CINV besides taking medication, and what life events and activities they missed out on when nauseated.	/	Gamed conversation, avatar, scenario of Daily living	Serious gaming for older adults should be an option to educate them about the home prevention of nausea and vomiting induced by chemotherapy.	Small population size. Participants became too focused on their own experience and insistent on specific aspects of their experience is included as a learning tool for others. A final limitation is that the CAB lacked diversity even though nurses identified populations with an excellent learning need
Maganty N., Ilyas M., Zhang N., Sharma A. (2018)	Pilot study	Florida, USA	Dermatology	To assess the effectiveness of a Game-based learning	Recruited in the Dermatology waiting room of Mayo Clinic. In	Participants were randomised into 3 groups: game, pamphlet and no intervention. Participants in the game group	Selection of MM image from a set of images that also contain benign nevi images.Correct benign	Game/pamphlet enjoyable: pamphlet 3.6 (1-5) and game 4.2 (1-5). The	Avatar (moles)	The results of this study support the efficacy of GBL educational tools for patients. Over half of	Small sample size, with 20 patients in each subgroup. Average education

				intervention, Tapamole, in improving recognition of the future of Melanoma compared to a written education intervention.	total 60 participants were recruited.	were linked to Tapamole and instructed to play the game. Participants in the pamphlet group were given a pamphlet on MM to read. Participants in the no-intervention group were not given any Melanoma education materials. A post-intervention survey asked all participants about their confidence in recognising Melanoma.	nevi images selcetion allows users to progress through the game. The MM images were retrieved from a Mayo Clinic image database.	sensitivity for MM recognition in the game group was 100% compared to 95% for the pamphlet group. The accuracy of the game group (60.6%) was similar to that of the pamphlet group (67.2%).		internet users search for health information online, suggesting the importance of having online health information. The GBL intervention was just as effective as the written intervention. More patients enjoyed the game-based intervention and would like to see it applied to other health issues.	level of participants being higher than that of the national average.
Reichlin L., Mani N., McArthur K., Harris A.M., Rajan N., Dacso C.C. (2011)	Mix-method	USA	Prostate	To assess usability if "Time After Time" by combining a survey and focus group study.	Men between the ages of 45 and 85 who were diagnosed with localised/early-stage prostate cancer after 1998 and before November 2007.	During the 6-month study period, a total of 13 participants attended 1 of 4 focus group sessions (3 groups of 3 participants and 1 group of 4 participants). Recruiting was concluded following the completion of these 4 focus group sessions, as focus group transcript analyses revealed a repetition of themes and responses indicating we had achieved a level of saturation appropriate for the preliminary testing of Time After Time	They collected quantitative measures of acceptance and usability from an 18-item instrument based on a 7-point Likert scale. This instrument was developed in line with user-centred game design principles, which use surveys or questionnaires to collect attitudinal data regarding participant views. Focus group discussions were recorded and transcribed verbatim. Using grounded theory as the basis for the analysis of focus group data [48], an audit committee of 5 researchers thoroughly reviewed the focus group session transcripts. Of the 5 researchers, 2 were experienced in coding and had training in qualitative analysis methods.	Acceptability, and usability of the game and its content. Quantitative: Likert scale. Qualitative: focus group.	Time After Time allows the user to explore potential side effects of 4 treatment options: radical prostatectomy, brachytherapy, external radiotherapy, and watchful waiting. For each treatment and each time period (immediately after treatment, 2 months after, and 12 months after), side effect card combinations are shown to the user. Each time a user reads a side effect card, he must rate it on a 5-point scale from 1, “no problem” to 5, “big problem.”	Initial research has made clear that game-based interactive decision aids for localised prostate cancer, like Time After Time, have the potential to fill an important need for newly diagnosed patients. Most study participants believed that Time After Time represents a valuable step in developing an appropriate decision tool for localised prostate cancer. Participants verified that the game meets the goals of increasing focus on HRQOL issues, generating questions for the patient’s healthcare team, and providing a new educational avenue to augment the patients’ participation in choosing a treatment for localised prostate cancer.	Small sample size. The sample was recruited from an active prostate cancer support group and its online community.
Loerzel V. W.; Clochesy J.M.; Geddie P.I. (2020)	Randomized Experimental study	Florida, USA	CINV	To examine the frequency and types of preventive and self-management behaviors of older adults with cancer. To report	80 older adults with cancer undergoing chemotherapy randomized in: Intervention group (n=38), control group (n=42). Patients' characteristics:	Inclusion criteria: participants aged 60 years or older, newly diagnosed with any cancer, on a three-week treatment cycle, receiving any chemotherapy agent with moderate-to-severe emetic potential, and proficient in English, and had a telephone. Exclusion criteria: previous diagnosis of cancer, prior treatment	The intervention consisted of two parts: playing the serious game (on an iPad in the treatment room before receiving the first chemotherapy treatment) and discussing the outcomes with the research nurse.	The intervention group kept more track of home preventive strategies (medication, diet, relaxation/distraction techniques) than in the control group. The control group	Avatar, simulated three-day scenario, levels, tutorial	Self-management behaviors may reinforce the importance of active prevention and management of CINV through serious game. The eSSET-CINV was shown to be acceptable and useful to older adults.	Small number of particpants. Difficult generlization because the participants were mostly whithe, educated older adults. CINV prevention and self-

				acceptability and usability data for the electronic Symptom SelfManagement Training–Chemotherapy-Induced Nausea and Vomiting (CINV) serious game.	range 60–84 years, most female (n = 59), White (n = 69), and non-Hispanic (n = 77), English as primary language (n = 78), college education or higher (n = 46), married or living with a partner (n=48), and retired (n = 64). Clinical: lung (n = 27), breast (n = 18), or uterine/endometrial (n = 15) cancer. Most participants received at least two chemotherapy agents (n = 59).	with chemotherapy, advanced or end-stage disease, palliative intent, visually or hearing impaired. Eligible participants were approached by the research nurse at the start of their first chemotherapy treatment appointment before receiving premedications.	In the serious game participants chose an avatar, watched a tutorial, and began to make self-management decisions (antiemetic medication, food-beverages, and other nonpharmacologic strategies). Simulated three-day scenario with various opportunities to make decisions.12–15 minutes estimated to complete game.	reported using more self-management behaviors than preventive behaviors for CINV overall.			management behaviors were not reported by all participants
Cosma G., Brown D., Shopland N., Battersby S., Symour-Smith S., Archer M., Khan M., Pockley A.G. (2016)	Evaluation study	Nottingham, UK	Prostate	To introduce PROstate Cancer Evalutation and Education (PROCEE) serious game to provide information and risk evaluation	29 participants voluntarily enrolled from an Afro-Caribbean community group.	The serious game prototype was installed on 20 tablets and each participant was given the opportunity to use the tablets to play the game from start to finish. Once all participants had used the game, they were placed into small focus groups, and were encouraged to provide feedback and suggestions for changes.	Play PROCEE serious game, the mean duration of completing the game is estimed 10-15 minutes	Feedback and suggestions to improve the game.	Game setting: barber salon target audience: 25-70 Number of levels: 8 Time needed to complete from start to finisch: 10-15 minutes.	PROCEE is serious game on prostate cancer which provided African-Caribbean men with information about the disease and also evaluates prostate cancer risk based on their profile and symptoms. The game is particularly useful to those users who are in a higher risk group due to their family history, age, ethnicity or symptoms. Enable patients to early recognise symptoms and seek medical advice.	Small sample size. African Caribbean men.
Thomas T. H.; McLaughlin M.; Hayden M.; Shumaker E.; Trybus J.,; Myers E.; Zabiegalski A.; Cohen, S. M. (2019)	Pilot study, co-design study	USA	Advanced cancer	To develop and assess the initial acceptability of a serious game to teach women with advanced cancer self-advocacy skills, including	18 adult females receiving treatment for a newly diagnosed advanced gynecological, lung, or gastrointestinal cancer at the community hospital in western	Two rounds of playtesting of "Strong Together" between February and March 2017 with 10 patients in total.	Play the game in which the avatar is named Angela and is a woman with advanced cancer, working mother trying to balance different social roles. The player must keep Angela healthy. The entire session is intended to last about 15–20 minutes. At the end of each	Patients after the game had: strong self-advocacy skills across all three dimensions (M = 4.55.0, SD = 0.8–0.9), low symptom severity (symptom prevalence: M = 2.8, SD = 1.7), and moderate to high quality of life (M =	Avatar, cartoon drawings, artwork, music-tone, story progression, realistic dialog and response options	The Strong Together serious game demonstrates the potential to assist patients in advocating for their needs and priorities	Small sample of women mostly with gynecological cancers. Need to expand the game to larger patient populations, to extend the results

				communication, decision-making, and social connectivity, to improve their quality of life with cancer.	Pennsylvania. Inclusion: capacity to speak and write in English.		session the player receives feedback about the choices she selected.	4.0, SD = 0.6). Patients enjoyed playing the Strong Together game (M = 5.9, SD = 1.0), felt like active participants (M = 5.5, SD = 1.6), and were socially engaged (M = 5.4, SD = 1.2). The game did not have a large impact on their mood (M = 3.7, SD = 0.8)			
Constantinescu G., Loewen I., King B., Brodt C., Hodgetts W., Rieger J. (2017)	Qualitative study	Canada	Head and Neck	1. To identify self-reported factors that influence adherence to conventional home therapy without a mobile device in Head and Neck Cancer patients and 2. To identify biofeedback	10 head and neck cancer patients (4 = F, 6 = M) recruited through tertiary care centres. Inclusion: difficulties with swallowing, previous experience with home-based unsupervised therapy following cancer treatment.	Patient semi-structured interviews. First part: identify the determinants of successful adherence to home-based swallowing therapy, select usefull information for app features. Second part: obtain reactions to designs for visual biofeedback. A semi-structured interview and convergent interviews were conducted	No intervention	- Perceptions on outcomes and progress<br>- Role of clinical appointments<br>- Cancer treatments<br>- Rehabilitation program<br>- Personal factors<br>- Connection	Coach, third person character, MyFitnessCoach (from Wii)	Through a biofeedback game, head and neck cancer patients were motivated and engaged	Small sample size. Convenience sample. Low adherence to home therapy.
Hee Jun Kim; Kim S. M.; Shin H.; Jang J.; Kim Y. I.; Han D. H. (2018)	Randomized controlled trial	Korea	Breast Cancer	To evaluate if using a mobile game in patient education increase drug compliance, decrease physical side effects of chemotherapy, and improve psychological status in breast cancer patients	Patient with IV stage breast cancer at Chung-Ang University Hospital, Korea, from September 2013 to September 2014. 76 females with metastatic breast cancer, aged 18-65 years (mean age 50.9), usingat least III-line palliative chemotherapy	3-week prospective trial. All participants received a combination of 4 chemotherapy (taxanes, anthracyclines, capecitabine, and cisplatin). Random allocation (interactive Web randomization system) in game group (n=36 education using mobile game play) or control group (n=40 conventional education). The mobile game (ILOVEBREAST) was installed on the smartphones of the intervention group. The control group received a 26 page education material (part 1= general infromation, part 2= individualized education)	Play the game "ILOVEBREAST" for >30 minutes a day, 3 times per week. Weekly phone interviews until the end of the study. An avatar is generated based on the patient's medical condition and treatment, the patients should make self-care decisions in the game.	High levels of satisfaction in the game group. Longer intervention time spent in the game group than in the control group (mean 22.2, SD 6.1 vs mean 5.5, SD 4.0 minutes a day). The mobile game group showed better drug adherence (mean 7.6, SD 0.7), lower rates of chemotherapy-related side effects (nausea, fatigue, numbness of hand or foot, and hair loss), better QoL during chemotherapy (mean 74.9, SD 3.5)	Avatar, alarm alerts, reward with "heart coins"	The serious game increase compliance to medication, knowledge on physical side effects, psychological support and QoL.	Small sample size, short study period. Adverse events assessed just in a qualitative way

								in game group. No significant differences in terms of depression and anxiety scales.			
Kondylakis H.; Bucur A.; Crico C.; Dong F.; Graf N.; Hoffman S.; Koumakis L.; Manenti A.; Marias K.; Mazzocco K.; Pravettoni G.; RenziC.; Schera F.; Triberti S.; Tsiknakis M.; Kiefer S. (2020)	Extensive literature review and co-design development process	Greece	Long-term cancer	To describe the "iManageCancer" project and to evaluate effectiveness and empowerment on a large-scale pilot for adults and a small-scale test for children with cancer using the ICT infrastructure	135 italian adults with cancer (88 woman with breast cancer and 47 men with prostate cancer) and 23 german families of children with cancer.<br>Inclusion criteria: adults with early stage of breast or prostate cancer, aged 18-75, active oncologic treatment, children with cancer and their parents.<br>Exclusion criteria: psychiatric comorbidities, metastatic disease, inability to fill questionnaires or sign the informed consent.	Random allocation to groups in IEO, Italy (for adults) and in the pediatric onco-ematology hospital in Germany (for children) during the test phase of the project. Patients in the eHealth intervention (eHI) group : were given a smartphone equipped with the iManageCancer platform at T0. 6-10 weeks of game . Patients in Informative Material (IM) group: standard care flow and standard material. After 6-10 weeks: same assessment as T0 for all patients. Three to five months after the beginning of the study, clinical performance indices were evaluated.<br><br>Evaluation tools: Resilience Scale for Adults, Mini-Mental Adjustment to Cancer scale (Mini-MAC), Profile of Mood States, Patient Health Engagement Scale used to analyze changes in patients' attitudes.	iManageCancer project includes intelligent apps, psychoemotional apps, apps for researchers and tailored serious games for adults and children.<br><br>Serious game for adults: lead the virtual character in making lifestyla, diet and exercise choices. possibility to play offline and share on social network<br><br>Serious game for children and adolescents: virtual travel in their vessel through the human body and fight various types of virtual cancer cells using multiple weapons gained by their supporters.	High quality and cost-effective care, motivation, patient empowerment, self-management, improve psycho-emotional status, improve understanding of the disease, involvment of caregiver, continuity of care.	Virtula character, exchange comments and score on social network, shooter and match game, weapons gained by other players	iManageCancer improved patient education, empowerment, resilience, reduced stress and psychological problems. No statistical analyses to demonstarte impact on children because of the small sample	Limited participation in the children study
Khalil G.E., Beale I.L., Chen M., Prokhorov A. V. (2016)	Three arm, single-blinded randomised controlled design	Houston, USA	Cancer Prevention	To evaluate and increase the perception of cancer risk and correct behaviour in healthy young adults survivors	44 young adults.	Random allocation in 3 groups: intervention group playing Re-Mission "High Challenge - HC" (n=85), intervention group playing Re-Mission "Low Challenge - LC" (n=81), and control group "No Challenge - NC" (n=50).<br>One week after completing a baseline survey, participants arrived at the intervention site and were randomly assigned to 1 of 3 conditions: LC, HC, or NC.<br>As an ethical consideration, after the 20-day follow-up, the NC group received information about Re-Mission and ways to access the game, if interested.	Participants were invited to start the first mission of the game and played for 35 minutes. Every time the players completed the mission and every time they lost in the game, they were asked to play it again, until the session was over.<br>Participants in all 3 conditions were invited to complete a survey immediately after implementation, 10 days later, and 20 days later.	ISB (Information Seeking Behaviour) was assessed at baseline, 10-day, and 20-day follow-ups. Perceived severity of cancer and perceived susceptibility to cancer were assessed at baseline, immediate post-test, 10-day, and 20-day follow-ups.<br>Other variables might affect play behaviour (eg, frequency of weekly gameplay,	Third person game, avatar (Roxxi, a nanorobot injected into the body to fight cancer cells). Levels, narrativr presentation, body scenario (hree-dimensional environment), shooting, fighting cancer cells, overcoming obstacles.	Re-Mission promoted a successfull medication adherence in young cancer patients. It increased perceived cancer severity, susceptibility and seeking of cancer-related information in young healthy adults.	Participants played only one time at "Re-Mission". Low retention rate (46.8%).

								perceived skills with video games, and perceived control over stress during gameplay).			
